# Pharmacokinetic evaluation of intravenous artesunate in adults with uncomplicated *falciparum* malaria in Kenya: a phase II study

**DOI:** 10.1186/1475-2875-13-281

**Published:** 2014-07-22

**Authors:** Qigui Li, Shon Remich, Scott R Miller, Bernhards Ogutu, Walter Otieno, Victor Melendez, Paktiya Teja-Isavadharm, Peter J Weina, Mark R Hickman, Bryan Smith, Mark Polhemus

**Affiliations:** 1 Division of Experimental Therapeutics, Walter Reed Army Institute of Research (WRAIR), 503 Robert Grant Avenue, Silver Spring, MD, 20910–7500, USA; 2 Kisumu Research Station, US Army Medical Research Unit, Kisumu, Kenya; 3 Department of Immunology and Medicine, Armed Forces Research Institute of Medical Sciences, Bangkok, Thailand

**Keywords:** Artesunate, Dihydroartemisinin, Pharmacokinetics, Intravenous, Uncomplicated *falciparum* malaria, Clinical trial

## Abstract

**Background:**

Alternatives to treatment for malaria treatment of travellers are needed in the USA and in Europe for travellers who return with severe malaria infections. The objective of this study is to show the pharmacokinetic (PK) profile of intravenous artesunate (AS), which was manufactured under good manufacturing practice (GMP) conditions, in adults with uncomplicated *falciparum* malaria in Kenya.

**Methods:**

The PK parameters of intravenous AS manufactured under current cGMP were evaluated after a single dose of drug at 2.4 mg/kg infused over 2 min in 28 adults with uncomplicated *Plasmodium falciparum* malaria. Plasma concentrations of AS and dihydroartemisinin (DHA) were measured using a validated liquid chromatography–mass spectrometry (LC-MS/MS) methodology. Pharmacokinetic data were analysed with a compartmental analysis for AS and DHA.

**Results:**

The results suggest there were no drug-related adverse events in any of the patients. After intravenous infusion, the concentration of the parent drug rapidly declined, and the AS was converted to DHA. AS and DHA showed mean elimination half-lives of 0.17 hours and 1.30 hours, respectively. The high mean peak concentration (C_max_) of AS was shown to be 28,558 ng/mL while the C_max_ of DHA was determined to be 2,932 ng/mL. Significant variability was noted in the PK profiles of the 28 patients tested. For example, C_max_ values of AS were calculated to range from 3,362 to 55,873 ng/mL, and the C_max_ value of DHA was noted to vary from 1,493 to 5,569 ng/mL. The mean area under the curve (AUC) of AS was shown to be approximately half that of DHA (1,878 ng·h/mL *vs* 3,543 ng·h/mL). The DHA/AS ratio observed was 1.94 during the one-day single treatment, and the AUC and half- life measured for DHA were significantly larger and longer than for AS.

**Conclusions:**

Intravenous AS can provide much higher peak concentrations of AS when compared to concentrations achieved with oral therapy; this may be crucial for the rapid elimination of parasites in patients with severe malaria. Given the much longer half-life of DHA compared to the short half-life of AS, DHA also plays a significant role in treatment of severe malaria.

## Background

Intravenous (IV) artesunate (AS) has been shown to completely inhibit parasite growth in infected human patients within two to four hours after dosing, and its active metabolite, dihydroartemisinin (DHA), is the only artemisinin derivative with activity against all asexual blood stage parasites [1]. AS treatment results in rapid parasite and fever clearance, and these effects have been mostly attributed to its rapid and extensive hydrolysis to DHA [2-4], the most active schizonticidal metabolite. *In vitro* bioassay tests have shown that the activity of DHA is similar to AS [5] and three- to five-fold more active than other artemisinin derivatives [6,7]. AS has been shown to be highly effective against multidrug-resistant *falciparum* malaria and for treatment of severe malaria in Vietnam, Thailand, China, and Myanmar, however, limited studies have been carried out in Africa [8]. Currently, there are three recommended treatments for severe and complicated malaria: AS, artemether (AM) and quinine (or quinidine) [9]. The Chinese manufacturer (Guilin Pharmaceutical Co Ltd, Shanghai, China) of the injectable AS that was used in most of the clinical trial studies recently improved its production process, with support from Medicine for Malaria Venture (MMV), and their product has achieved World Health Organization (WHO) recognition. Intravenous quinine or quinidine, however, is still used in Europe and the USA as the main barrier for the use of intravenous AS is the absence of an approved product that is manufactured under good manufacturing practices (GMP) and is legally available in these regions for patient treatment [9].

Following recent clinical studies with severe malaria patients, IV AS was shown to have the highest success for treatment of malaria with a low incidence of adverse events [10-12]. The two largest trials ever conducted for severe malaria in endemic regions showed that, in both adults and children, treatment with IV AS yields better outcomes than treatment with IV quinine. The mortality rate among quinine-treated patients was shown to be 22 and 10.9% in the Southeast Asia Quinine Artesunate Malarial Trial (SEAQUAMAT) and Africa Quinine Artesunate Malarial Trial (AQUAMAT) studies, respectively, and the mortality rate among AS-treated patients was shown to be 15 and 8.5%, respectively. When comparing the mortality rates achieved with treatment of these two drugs, AS reduced mortality by 35% in the SEAQUAMAT trial and 22.5% in the AQUAMAT trail. Patients with hyperparasitaemia (>10% of red blood cells (RBC) experienced a significantly greater treatment effect after AS treatment than patients that did not have hyperparasitaemia. IV AS was also shown to be better tolerated, safer and easier to use than quinine. The life-saving benefit of AS for treatment of severe malaria was recognized by the WHO in 2010, and since then IV AS has been the initial treatment of choice for patients with severe *falciparum* malaria infections [11,12].

Alternatives to treatment for severe malaria are needed in the USA and Europe for treatment of severe malaria. While nations in the Third World can choose to use AS preparations such as the Guilin AS product for treatment of disease, there are issues with the use of this AS product in First World countries where the Guilin AS formulation cannot be sold legally. The objective of this study is to show the pharmacokinetic (PK) profile of IV AS in Kenyans infected with uncomplicated *falciparum* malaria. The PK estimates will be also used to compare with the PK profiles observed in populations of healthy adults administered AS in another manuscript. The overall goal in the present study was to provide a well-validated, efficacious, and safe product for the treatment of severe malaria licensed by the US Food and Drug Administration (FDA) available for use in the USA and by US soldiers who encounter severe malaria infections.

## Methods

### Chemicals

The bulk artesunic acid (4-(10’dihydro-artemisinin-oxymethyl) succinate) substance was purchased from Knoll AG (Switzerland). BASF Pharmaceuticals rebottled it from the original company under GMP conditions. The clinical trial AS (Batch #: 14462–16) was tested for sterility and short-term stability. The formulation was contained in sterilized bottles with 110 mg artesunic acid per bottle. The injection buffer for AS was manufactured as a GMP phosphate salt with 0.3 M PBS (pH 8.0). The product was prepared for administration by reconstitution in sodium phosphate buffer prior to infusion, which took place within 1 hr after solution was prepared due to a precipitation in 3–4 hours at room temperature.

### Subject background

All subjects gave written informed consent prior to the commencement of any study procedure (e.g, blood smear). Overall, 30 adult subjects were enrolled, 20 males and ten non-pregnant females, aged 18–65 years, with uncomplicated *Plasmodium falciparum* malaria and body weights of 42–85 kg. The subjects of this study resided in a malaria-endemic region of Kenya and in districts surrounding the Province of Nyanza. The majority of the population in these areas is considered semi-immune, however, malaria attack rates in the city are low, and many adults in this area may well have waning immunity. The prevalence of malaria in Nyanza Province has been shown to range from 25 to 45%.

Adult subjects were recruited with signs and symptoms of malaria who were seen at the Nyanza Medical Centre, Kisumu or one of its neighbouring health clinics (Kisumu District Hospital in Kisumu; Nyahera Health Centre, Rabuor Dispensary, in Kisumu, and Kombewa Subdistrict Hospital), Nyanza Province. Local clinicians made a provisional clinical diagnosis of malaria, and these patients were subsequently referred to the hospital laboratory for microscopic examination of blood smears or rapid malaria antigen tests. Those subjects from the hospital laboratory who had not initiated therapy after a positive test were recruited for this study.

The screening process for subject participation consisted of questionnaires and clinical examinations including medical history, physical examination, and laboratory analyses. Phlebotomy was performed to obtain samples for laboratory testing and malaria blood films. A clean-catch urine specimen was also collected for urinalysis and pregnancy testing, as applicable. Baseline safety testing included both clinical chemistry and haematology testing. Analyses included blood urea nitrogen (BUN), creatinine, aspartate aminotransferase (AST), alanine aminotransferase (ALT), total and direct bilirubin, reticulocyte, and a complete blood cell (CBC) count with differential assessment. Ultimately, enrolment determinations consisted of compliance with inclusion and exclusion criteria, including a diagnosis of uncomplicated *P. falciparum* malaria with at least 200 parasites per μL confirmed by malaria blood smear.

### Study design

This was a Phase II, open-label, non-randomized PK clinical trial of IV AS. Subjects completed an outpatient visit at the study centre for confirmation of study eligibility and baseline electrocardiogram monitoring. All subjects received an identical dose of the test article: injectable IV AS daily for two days consisting of 2.4 mg/kg delivered over 2 min. Safety, PK and efficacy assessments occurred intermittently with follow-on standard therapy with a three-day course of Malarone® to ensure cure. After completion of each injection, the study nurse examined how well AS injection was tolerated by performing inspection of the injection site and subject response. Normal activities, excluding strenuous exercise, were permitted four hours after dosing. The clinical trial identifier in ClinicalTrials.gov is NCT00298610 [13]. The human use protocol numbers for this Phase II trial are Uniformed Services University of the Health Sciences (USUHS) #G183RW; Walter Reed Army Institute of Research (WRIAR) #1128; Human Subjects Research Review Board (HSRRB) #A13331, and the Kenya Medical Research Institute (KEMRI) #917.

### PK and finger-stick sampling

PK sampling was performed using a flush saline technique from an IV line or phlebotomy if the IV line was compromised. Samples were collected on day 1 approximately 10 min before dosing (0 hours), and at the following nominal time points after dosing: 5, 10, 20, and 40 min, and 1, 2, 4, and 6 hours. The blood (6 ml) will be collected from each subject via a cannula or by repeated venipuncture (opposite arm from infusion) into lithium heparin tubes. Actual time points were obtained within ± 20% of the stated time points, and the plasma samples were immediately stored at approximately −70°C after collection, and shipped to the Armed Forces Research Institute of Medical Sciences (AFRIMS) Bangkok, Thailand on dry ice, for analysis. Finger-stick sampling was used for any malaria blood films collected at time points not requiring phlebotomy.

### Treatment and monitoring

Subjects were treated and monitored as outpatients or inpatients as deemed necessary by the subject, study staff and the principal investigator. Subjects managed as outpatients were permitted to leave the study site after the first dose of study drug (day 0) and sampling (approximately eight hours). Subjects were required to return the following day for scheduled follow-up.

### LC-MS assay

An LC-MS method for the quantitation of AS and DHA (100 μL) in human plasma was validated in a range from 1.4-1,153 ng/mL for AS and from 1.1-853 ng/mL for DHA. The analytes were extracted from human plasma after protein precipitation using two volumes of ice-cold acetonitrile. After centrifugation at 10,000 rpm, the clear supernatant extracts were analysed on a single quadrupole Mass Spectrometer (Micromass ZQ, MM1, Waters Corp, Milford, MA, USA) in the positive ion electrospray ionization (+ESI) mode. The AS and DHA compounds were monitored using single ion recording. The LC-MS with a reversed phase column (XTerra MS C18, 3.5 μm, 2.1 × 50 mm) and a pre-column of the same material (2.1 × 10 mm) was mounted on a liquid chromatography system (Alliance 2695, Waters Corp, Milford, MA, USA). The method was performed a gradient elution with the following mobile phases: A) 6.25 mM ammonium acetate in water (pH 4.5) and B) 100% acetonitrile gradient from 20 to 40% in ammonium acetate buffer in 9 min at the flow rate of 0.4 mL/min. The ammonium-adducts of DHA were detected at a mass to charge ratio (m/z) of 302 and eluted in 5 min, while adducts of AS were detected at a mass to charge ratio of 402 and eluted in 6 min. The total analysis time for both compounds was 12 min.

In these methods, the intra-assay and inter-assay accuracy and precision (n = 6) had an inaccuracy rate of ± 8.4% and coefficient of variations (CVs) of ≤7.9% at 19.2/14.2 (50), 192/142 (500), 961/711 (2,500) and 1,153/853 (3,000) ng/mL (nM) for AS/DHA. The intra-assay and inter-assay inaccuracy and precision were within ± 6.0 and ≤17.6%, respectively [14]. Standard curve and quality control (QC) samples were generated by spiking interference-free human plasma samples with known amounts of AS, DHA and internal standard. The drug concentrations of the QC samples chosen were within the range of the standard curve, and included a low (<3 × LLOQ), medium, and high QC levels. The lower limit of quantitation (LLOQ) was 1.44/1.07 (3.75) ng/mL (nM) for AS/DHA. Any out-of-trend concentration values were re-analysed with the average of three repeated values used in PK parameter determination. Calibration standards and QC samples were analysed to evaluate the performance of the assay and were the same as the validated method previously described [14].

### Pharmacokinetic analysis

In order to compare only the clinically relevant AS concentrations, a compartmental PK analysis was based on a short-term (2 min) IV infusion on individual patient level in accordance with a previous PK evaluation [15,16]. The disposition of injectable AS was best described by a two-compartment model with a rapid initial distribution phase after IV administration. The input of the drug was assumed to follow zero-order kinetics, and elimination from the central compartment occurred with first-order kinetics. compartmental analysis (CA) of concentration-time data for AS was performed by using the Phoenix-WinNonlin software Version 6.3 (Pharsight Corp, Mountain View, CA, USA) with an IV infusion programme. The first-order method with logarithmic transformation of all drug-concentration data was used throughout.

The drug plasma concentrations at the end of IV infusion (*C*_max_) were calculated from the corresponding model equations (IV-infusion model, WinNonlin) at the respective time points. The AUC_inf_ was estimated by direct integration of equation with CA modeling from zero to infinity. The total clearance (CL) is calculated with dose divided by total AUC. The *C*_max_ and the time to maximum drug concentration (T_max_) were obtained from the PK modeling. The predicted AS concentration at the end of the infusion (2 min), however, was calculated by compartmental modelling. The values of the following PK parameters were derived by a compartmental method. The elimination half-life (t½), mean residence time (MRT), the AUC from time zero to the last sampling time (AUCt), the AUC from time zero extrapolated to infinity (AUC_inf_), total systemic clearance (CL), and the volume of distribution during steady state (*V*_ss_) were obtained by PK modeling.

Previous PK analysis [15] and PK estimates in the present study confirmed that the PK parameters of DHA are similar when using the non-compartmental analysis (NCA) and CA analyses, suggesting that PK modelling of DHA is model-independent. Thus, the both methods are suitable for DHA. The values of the following PK parameters were derived by a compartmental method with the C_max_, t½, MRT, AUC_inf_, relative total systemic clearance (CL/F), and the volume of distribution during steady state (*V*_ss_/F). The AUC_inf_ ratios of AS to its metabolite (DHA) were calculated to determine exposure to any metabolite compared with the parent drug.

## Results

IV AS manufactured to be cGMP compliant was administrated to 30 adult patients. PK samples were not collected for two subjects (Two cases have been excluded due to the insufficient data). Efficacy and safety evaluations will be discussed in detail in a separate manuscript.

### Safety and tolerability

In this study, 30 patients with uncomplicated *P. falciparum* malaria were treated with an intravenous infusion of AS (2.4 mg/kg given over 2 min) daily for two days. For each subject, adverse events (AEs) were recorded throughout the post-dosing period. Multiple doses of this formulation of AS administered intravenously at a dose of 2.4 mg/kg daily for two consecutive days were well tolerated in all patients. No dose related toxicity was found for AS at the doses studied. There were no subject dropouts for AEs or other treatment-related issues. The dose-related decrease in reticulocyte count was noted that reached its lowest point four days after dosing and returned to normal by study day 7 in most cases. There were no other clinically significant laboratory abnormalities detected. No deleterious haemodynamic or electrocardiography (ECG) effects were seen. A transient, reversible sensation of altered or unusual taste, which lasted less than 30 min in all cases, was associated with intravenous dosing of AS. All remaining side effects were generally mild and all were reversible.

### Pharmacokinetics of AS and DHA

**Figure 1:**
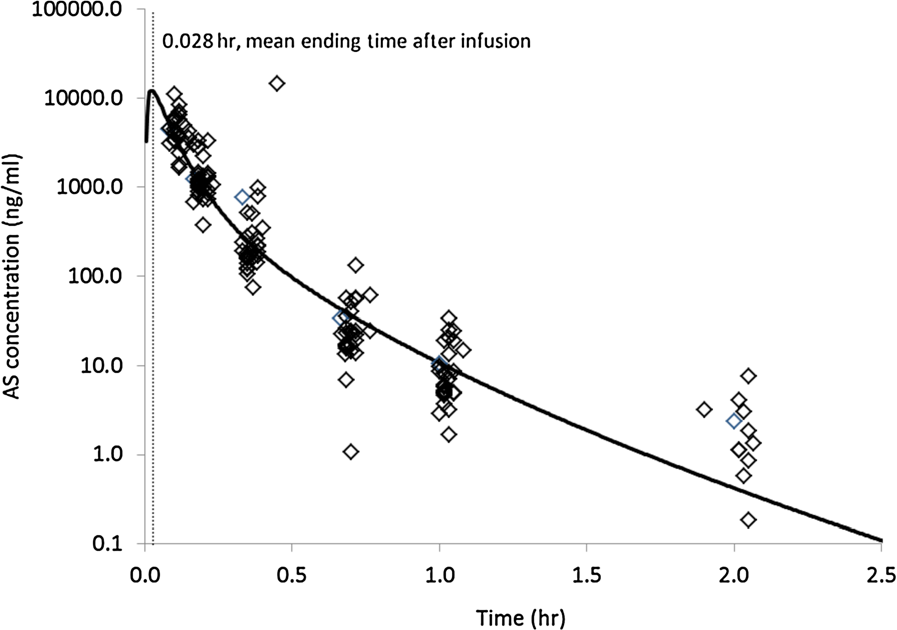
**Concentration-time profile of artesunate (AS) in plasma measured by LC-MS (empty diamond) and mean concentration curve (solid-line) fitted by the compartment analysis with intravenous infusion model following 0.028 hr intravenous infusion (2 minutes) with cGMP injection AS at dose of 2.4 mg/kg in adult patients with uncomplicated *falciparum *malaria (n** **=** **28).**

PK evaluations of AS for the 28 subjects in this study were completed using compartmental model analysis. The compartmental PK analysis performed resulted in a two-compartment model of IV infusion with first-order elimination, which best fit the set of observations [15,16]. The plasma AS concentration and the final PK parameter estimates for AS after IV infusion of 2.4 mg/kg AS based on a two-compartment model are shown in Figure 1 and Table 1, respectively. After the short-term infusion of AS (0.028 hours), the mean C_max_ of AS was shown to be 28,558 ng/mL at the ending time of the infusion for the study population. The comparison results support the suggestion that PK analysis of AS may be performed only with compartment modelling [15]. After the injection of 2.4 mg/kg of AS, the mean AUC obtained with this dose was 1,878 ng·h/mL. The elimination half-life for AS was ranged from 0.09 to 0.25 hours with a mean elimination half-life of 0.17 hours. The MRT of AS was shown to be only 0.07 hours following AS infusion. The CL and *V*ss were calculated to be 1,728 mL/h/kg and 139 mL/kg, respectively (Table 1).

**Table 1:** **Mean pharmacokinetic parameters of artesunate** (**AS**) **and its active metabolite dihydroartemisinin** (**DHA**) **after pharmacokinetic modelling by using compartment analysis with IV**-**infusion fitting for AS following single intravenous infusion** (**2 minutes**) **at single dose of 2.4 mg**/**kg in the adults with uncomplicated *falciparum *malaria in Kenya** (**n** = **28**)

**PK parameters artesunate**	**CA IV-infusion (PK model 9)**	**CV (%)**	**PK parameters dihydroartemisinin**	**CA analysis (PK model 14)**	**CV (%)**
C_max_ (ng/mL)	28,558 ± 28,531	99.8	C_max_ (ng/mL)	2,932 ± 850	28.9
T_max_ (h)	0.028 ± 0.015	54.6	T_max_ (h)	0.18 ± 0.06	30.0
AUC_inf_ (ng · h/mL)	1,879 ± 1,190	63.4	AUC_inf_ (ng · h/mL)	3,543 ± 989	27.9
t_1/2__distribution_ (h)	0.05 ± 0.01	15.2	t_1/2__distribution_ (h)	0.13 ± 0.05	38.5
t_1/2__elimination_ (h)	0.17 ± 0.08	47.4	t_1/2__elimination_ (h)	1.30 ± 0.34	26.5
CL (mL/h/kg)	1,728 ± 983	56.9	CL/F (mL/h/kg)	731 ± 205	28.0
Vss (mL/kg)	139 ± 124	89.2	Vss/F (mL/kg)	1,048 ± 250	23.8
MRT (h)	0.07 ± 0.02	30.9	MRT (h)	1.61 ± 0.44	27.3
Ratio of AUC_DHA/AS_				1.94 ± 1.16	59.8
Ratio of C_max__AS/DHA_				10.24 ± 7.61	74.3
Ratio of t_1/2__DHA/AS_				7.65 ± 3.16	41.3

Data are presented as arithmetic mean (CV%); *CA* = compartment analysis (Phoenix-WinNonlin Version 6.3 with compartment model 9, IV-infusion for AS and model 14 for DHA); F = bioavailability; *MRT* = mean residence time.

**Figure 2:**
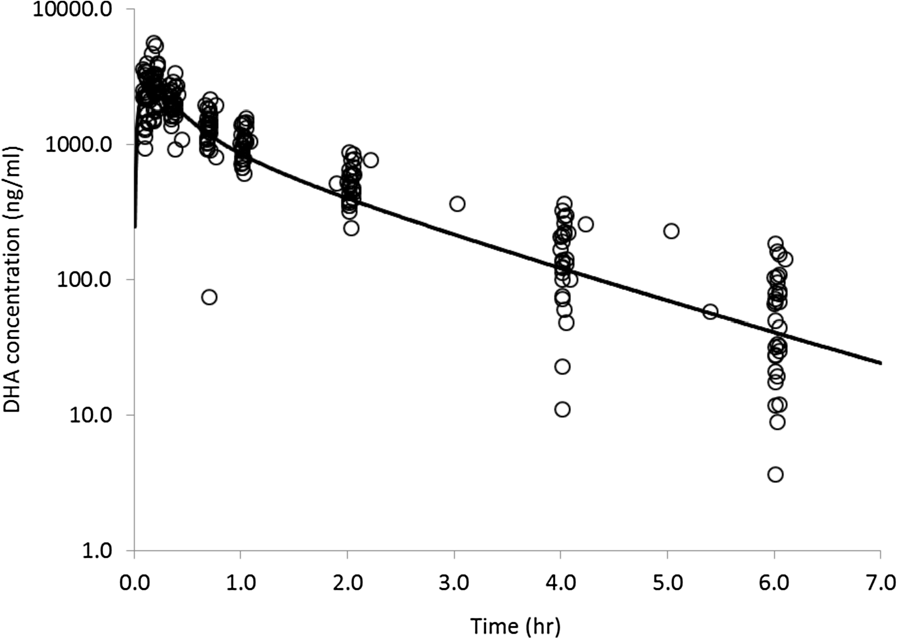
**Concentration-time profile of dihydroartemisinin (DHA) in plasma measured by LC-MS (empty cycle) and mean concentration curve (solid-line) fitted by the compartment analysis following single intravenous infusion with cGMP injection AS at dose of 2.4 mg/kg in adult patients with uncomplicated *falciparum *malaria (n** **=** **28).**

The plasma concentrations of DHA after short-term infusion of a single dose of 2.4 mg/kg are shown in Figure 2. Table 1 shows the mean parameters derived in a first approach using the CA modeling. The mean *C*_max_ and AUC_inf_ for DHA were shown to be 2,932 ng·/mL and 3,543 ng · h/mL, respectively, in the AS-treated patients. The mean T_max_ was shown to range from 0.12 - 0.28 hours, and the mean elimination t½ was shown to be 1.30 hours. The CL/F and *V*ss/F were shown to be 731 mL/h/kg and 1,048 mL/kg, respectively.

### Variable PK profiles of AS and DHA

In the 28 malaria patients evaluated in this study, the peak concentrations (*C*_max_) of AS following IV infusion ranged from 3,362 to 55,873 ng/mL, and the AUC of AS ranged from 477 to 6,434 ng · h/mL, suggesting a large inter-individual variability in these PK parameters. Similarly, the *C*_max_ of DHA ranged from 1,493 to 5,569 ng/mL, and the AUC values calculated ranged from 2,024 to 5,424 ng · h/mL.

The concentration of DHA, which was converted from AS, was also shown to be highly variable in the 28 patients evaluated. The ratio of AUC_DHA/AS_ ranged from 0.72 to 4.16 (Table 1). In addition, the AUC of DHA was significantly higher than the AUC of AS with a mean AUC_DHA/AS_ ratio of 1.94, however, the peak concentration of AS was much higher than that of DHA with a mean *C*_max__AS/DHA_ ratio of 10.24. DHA was also shown to have a 7.65-fold longer elimination half-life than AS.

## Discussion

IV AS has been used to treat patients with both uncomplicated *P. falciparum* and severe malaria for more than 25 years [11]. Artesunate has been shown to be highly effective against multidrug resistant *falciparum* malaria and for treatment of severe malaria in Asia. However, the main barrier for the use of intravenous AS in Western countries is the absence of an approved product manufactured under GMP. A major concern that has been expressed is that the currently available AS products available for use are not available in Western countries for treatment of travellers with severe malaria because they are not licensed for use in either Europe or the USA. While there is no evidence in clinical trials conducted to date that the absence of GMP-produced AS translates into reduced efficacy, there are significant long-term benefits to patient care associated with implementing a licensed GMP artesunate product that is available for treatment of travellers with severe malaria who cannot be given an unlicensed product. GMP AS is currently under development, both in China and in the USA, for treatment of *falciparum* malaria [17].

The two available methods used for determining PK parameters of AS and DHA are the compartmental (model-dependent) and non-compartment (model-independent) PK analytical techniques [18]. PK evaluation of IV AS with a short-term IV infusion, however, requires compartmental analysis. When using NCA for AS PK parameter modelling, the AUC calculation for AS will be adversely perturbed due to the loss of accurate assessment of *C*_max_ at the end of the 2-min IV infusion. In previous published reports, the AUC of AS calculated using NCA underestimated the AUC parameter by 28-49% when compared to CA analysis [15,16]. Similar underestimation of AUC was also shown in this study. Therefore, the CA analysis model is capable of accurately determining the PK parameters of both AS and DHA in this study. For further evaluation of PK modeling of intravenous AS administered by infusion, a specific manuscript is being prepared based on the PK data from three clinical trials utilizing a variety of PK software packages.

In this study and as previously reported, artesunate is rapidly converted to DHA [9]. DHA was detected in all patients’ plasma up to six hours after administration, while the parent drug (AS) was undetectable at time-points as early as one to two hours after each short-term infusion. Although the plasma exposure of DHA was increased when compared to AS with an AUC_DHA/AS_ ratio of 1.94, the peak concentration of AS was shown to be much higher than DHA with a *C*_max__AS/_*C*_max DHA_ ratio of 10.24.

In this study, DHA was shown to have 7.65-fold longer half-life than that of AS. Therefore, the efficacy of AS has been attributed both to its own intrinsic activity and to the activity of its principle metabolite, DHA [2-4]. The high peak concentrations achieved after dosing patients intravenously with AS provides a significant advantage over other anti-malarial drugs to achieve rapid killing of malaria parasites. A number of pharmacokinetic and pharmacodynamic evaluations have shown that the rapid efficacy of artemisinins is principally due to the elevated *C*_max_ achieved after drug administration, and further studies have shown that AS is superior to other artemisinin analogues in terms of the very high *C*_max_ observed following oral or IV administration [19]. Other PK parameters, such as the drug exposure level (AUC), and drug exposure time (half-life) have been shown to be of lesser importance [19,20]. A number of studies have also shown the rapid pharmacodynamic effects of these drugs in rapidly decreasing the parasite biomass after administration [21].

Significant inter-individual variability in AS PK parameters was shown in this study with coefficients of variation (CV) of 47.4-99.8% (Table 1), which is much higher than the CVs observed for DHA, which ranged from 23.8-38.5%. One particular patient in previous study showed the highest peak concentration of AS at 55,873 ng/mL, which was 16.6-fold more than the patient with the lowest AS concentration (3,362 ng/mL). The currently used therapeutic doses of injectable AS administered for treatment of severe and complicated malaria are in the range of 2–2.4 mg/kg. This regimen will produce a *C*_max_ of AS ranging from 130–16,149 ng/mL in adult patients, demonstrating the significant variability (about 124-fold between different trials) following intravenous AS administration (Table 2, Refs. [22-28]). One could speculate that the substantial variability is a function of imprecision around the duration of the infusion dose, the relative imprecision around the plasma sampling, the rate of AS converted to DHA, and various other individual patient factors.

**Table 2:** **The main pharmacokinetic parameters of artesunate** (**AS**) **and dihydroartemisinin** (**DHA**) **following intravenous** (**IV**) **administrations in malaria patients and volunteers**

					**Artesunate (AS)**				**Dihydroartemisinin (DHA)**			**Ratio of AUC**_**DHA/AS**_	**Refs**
**Patients**	**Where**	**Cases**	**Date**	**Route**	**Dose**	**C**_**max **_**ng/mL**	**AUC ng** **·** **h/mL**	**t**_**1/2**_**min**	**C**_**max **_**ng/mL**	**AUC ng** **·** **h/mL**	**t**_**1/2**_**min**		
Children	Gabon	10	2002	IV	2.4 mg/kg	29,683	3,752	1.5	3,012	3,325	20.7	0.89	[22]
Children	Ghana	9	2001	IV	2.4 mg/kg				1,593	1,708	31.8	-	[23]
Adults	Vietnam	13	1998	IV	120 mg/person	11,343	1,146	2.7	2,646	2,378	40.2	2.08	[24]
Adults	Vietnam	11	2002	IV	120 mg/person	16,149	1,038	3.2	2,760	2,873	59.0	2.77	[25]
Adults	Thailand	2	2000	IV	2 mg/kg	-	1,056		-	3,999	-	3.79	[26]
Adults	Vietnam	12	1998	IV	120 mg/person	13,688	877	2.2	2,193	1,846	36.7	2.10	[2]
Volunteer	Vietnam	10	2001	IV	120 mg/person	-	846	2.6	1,508	1,365	53.0	1.61	[27]
Adults	Vietnam	8	2001	IV	120 mg/person	-	-	2.3	2,423	2,081	40.0	-	[3]
Adults	Vietnam	6	2001	IV	120 mg/person	-	-	4.3	2,537	2,565	64.1	-	[28]
Adults	Vietnam	10	2001	IV	240 mg/person	-	-	3.2	912	5,586	46.2	-	[27]
Adults	Thailand	17	2006	IV	2.4 mg/kg	130	49	13.2	605	418	20.4	8.53	[28]
Volunteer	USA	6	2009	IV	2 mg/kg	19,420	1,595	14.4	1,456	1,821	78.6	1.14	[15]
Volunteer	USA	6	2012	IV	2 mg/kg	28,411	2,051	9.6	1,735	2,121	104.4	1.03	[16]
Adults	Kenya	28	2014	IV	2.4 mg/kg	28,558	1,878	10.2	2,932	3,543	78.0	1.94	Present

The drug measurements are by using HPLC-ECD or LC-MS methods, and some drug evaluations by using bioassay are not selected in this summary.

*PNG* = Papua New Guinea.

The dose of intravenous AS currently used for clinical use was designed in accordance with prior experience of physicians over the last 25 years who were concerned with neurotoxicity and dose-dependent haemotoxicity and thus, the dose was limited to 2–2.4 mg/kg to prevent a submaximal anti-malarial effect, and to sustain a minimum curative rate >90% [29]. The lower peak concentrations achieved in some patients, however, especially those who have severe and complicated malaria associated with hyperparasitaemia of >5% may result in clinical failure. In the SEAQUAMAT and AQUAMAT clinical trial studies, IV AS was shown to be superior to quinine, and patients with hyperparasitaemia had a significantly greater treatment response to AS than in patients without hyperparasitaemia [9-12].

In theory, patients with severe malaria require a peak concentration of AS ranging from 7,500-30,000 ng/mL to efficaciously exterminate malaria parasites where parasitaemia exceeds 5% [30]. This calculation is based on the drug concentration achieved at equilibrium after oral AS administration of 1,250 ng/mL (AS plus DHA), which was required to cure patients infected with malaria who had a parasitaemia of 1%. The estimation of patients with severe malaria would require a peak plasma concentration of AS plus DHA at least 7,500 ng/mL to effectively reduce the parasite biomass in patients with severe malaria given previous data and the data from other trials. Accordingly, dosing of AS at 2.0 - 2.4 mg/kg intravenously will likely not satisfactorily distribute an efficacious drug level to every erythrocyte (infected and uninfected) in each patient with hyperparasitaemia. When taking into account the large variability in PK and PD between individuals, a larger dose will be required to ensure that a maximum effect is obtained in all patients. The patients with hyperparasitaemia are effectively treated is to increase the intravenous loading dose to a higher level in the range of 4–8 mg/kg, which should be well tolerated given other studies showing single and multiple injections of AS in volunteers up to a dose of 8 mg/kg dose were exceedingly well tolerated [15,16]. However, further trials are needed to test this dose and the frequency of IV AS treatments at that dose required to achieve cures of patients with severe malaria patients.

## Conclusion

The study presented here underlines the need for appropriate PK analysis of AS and DHA following intravenous infusion. Injectable AS is a superior anti-malarial agent yielding very high peak plasma concentrations over a very short exposure time. The higher peak level serves to eliminate parasites rapidly, and the short exposure time aids in avoiding fatal neurotoxicity [31]. Previous data from other studies have shown that administration of injectable AS at doses up to 8 mg/kg is safe [15,16], and given the large inter-individual variability associated with AS administration, use of higher doses may increase the probability of success in patients with hyperparasitaemia associated with severe malaria [20,32].

## Competing interests

The authors have declared that they have no competing interests.

## Authors’ contributions

SR and MP conceived the study. SR, BO and WO conducted the clinical trial study. PT, VM and BS conducted LC/MS/MS study. PW, SM and MH reviewed and edited this manuscript. QL analysed PK data and wrote the manuscript. All authors read and approved the final manuscript.
